# A Review and Bibliometric Analysis of Studies on Advances in Peripheral Nerve Regeneration

**DOI:** 10.7759/cureus.69515

**Published:** 2024-09-16

**Authors:** Billy McBenedict, Wilhelmina N Hauwanga, Gabriel Escudeiro, Dulci Petrus, Barakat B Onabanjo, Chukwuwike Johnny, Mohamed Omer, Amoolya R Amaravadhi, Asaju Felix, Ngoc B Dang, Lorena Adolphsson, Bruno Lima Pessôa

**Affiliations:** 1 Neurosurgery, Fluminense Federal University, Niterói, BRA; 2 Cardiology, Faculty of Medicine, Federal University of the State of Rio de Janeiro, Rio de Janeiro, BRA; 3 Family Medicine, Directorate of Special Programs, Ministry of Health and Social Services, Windhoek, NAM; 4 Research and Development, Montefiore Medical Center, Wakefield Campus, New York City, USA; 5 Family Medicine, Life Point Medical Centre, Abuja, NGA; 6 Internal Medicine, Sulaiman Al Rajhi University, Ar Rass, SAU; 7 Internal Medicine, Malla Reddy Institute of Medical Sciences, Hyderabad, IND; 8 General Practice, Dorset County Hospital, Dorchester, GBR; 9 Nursing, College of Health Sciences, VinUniversity, Hanoi, VNM; 10 Neurosurgery, Fluminense Federal University, Niteroi, BRA

**Keywords:** bibliometric analysis, functional recovery, peripheral nerve regeneration, research trends, schwann cells

## Abstract

Peripheral nerve injuries (PNIs) pose significant clinical challenges due to their complex healing processes and the often incomplete functional recovery. This review and bibliometric analysis aimed to provide a comprehensive overview of advancements in peripheral nerve regeneration research, focusing on trends, influential studies, and emerging areas. By analyzing 2921 publications from the Web of Science Core Collection, key themes such as nerve regeneration, repair, and the critical role of Schwann cells were identified. The study highlights a notable increase in research output since the early 2000s, with China and the United States leading in publication volume and citations. The analysis also underscores the importance of collaborative networks, which are driving innovation in this field. Despite significant progress, the challenge of achieving complete functional recovery from PNIs persists, emphasizing the need for continued research into novel therapeutic strategies. This review synthesizes current knowledge on the mechanisms of nerve regeneration, including the roles of cellular and molecular processes, neurotrophic factors, and emerging therapeutic approaches such as gene therapy and stem cell applications. Additionally, the study revealed the use of nanotechnology, biomaterials, and advanced imaging techniques, which hold promise for improving the outcomes of nerve repair. This bibliometric analysis not only maps the landscape of peripheral nerve regeneration research but also identifies opportunities for future investigation. This study has some limitations, including reliance on the Web of Science Core Collection, which may exclude relevant research from other databases. The analysis is predominantly English-based, potentially overlooking significant non-English studies. Citation trends might be influenced by shifting research priorities and accessibility issues, affecting the visibility of older work. Additionally, geographical disparities and limited collaboration networks may restrict the global applicability and knowledge exchange in this field.

## Introduction and background

The nervous system consists of the central nervous system (CNS), including the brain and spinal cord, and the peripheral nervous system (PNS), which comprises peripheral nerves. Upon injury at the PNS, a series of events ensues, starting with axonal breakdown and the formation of amorphous debris that serves as a substrate for nerve regeneration [1]. Subsequently, Schwann cells differentiate into a pro-repair state, and macrophages are recruited to clear cellular debris and release neurotrophic factors [1]. Traumatic peripheral nerve injuries (PNIs) encompass various conditions that damage one or more peripheral nerves, often resulting in the loss of motor or sensory functions. Trauma is a leading cause of PNIs, particularly prevalent among young individuals, with an estimated incidence ranging from 1.46% to 2.8%, especially affecting the upper extremities [2]. These injuries can arise from various causes, including trauma, surgical procedures, and certain medical conditions, leading to sensory and motor deficits that profoundly impact the quality of life. Unlike the CNS, the PNS possesses a remarkable capacity for regeneration [2].

Despite this inherent regenerative capacity of peripheral nerves, functional recovery is often incomplete and can be slow, particularly in cases of severe injuries or when the nerve gap is substantial [3]. This has driven extensive research into enhancing nerve regeneration through various strategies, including surgical interventions, biomaterials, pharmacological agents, and cell-based therapies. Despite a well-understood nerve regeneration pathway, treatment remains challenging, as no intervention significantly enhances the nerve repair process to achieve complete sensory or functional recovery [4-6]. Treatment options for peripheral nerve lesions include surgical techniques such as allografts, autografts, microsurgery, and guidance conduits. Each technique has its own advantages and disadvantages, depending on the extent, location, and severity of the nerve injury [5].

Understanding the mechanisms underlying peripheral nerve regeneration and translating this knowledge into effective therapeutic strategies is a critical area of ongoing research. By harnessing the regenerative potential of the PNS and leveraging cutting-edge technologies, there is hope for improving outcomes for individuals suffering from peripheral nerve injuries, ultimately enhancing their quality of life and functional independence. This review aimed to synthesize the current information regarding peripheral nerve regeneration and to evaluate the scientific output and trends in this field using a bibliometric analysis. This bibliometric analysis comprehensively reviewed the literature on peripheral nerve regeneration, including keywords such as treatment, therapy, nerve grafts, nerve conduits, and stem cell therapy. By examining trends and patterns in scientific publications, this study sought to provide insights into the evolution of research in peripheral nerve regeneration.

## Review

Materials and methods

Data Sources and Search Strategies

The scientific papers were collected from the Web of Science Core Collection (WoSCC) on July 10, 2024. To accomplish a thorough analysis of the publications and prevent any fluctuations in citation counts, there were no restrictions on the publication year and the search and download were conducted on the same day. This study included articles, review articles, proceeding papers, early access, book chapters, and publications with expressions of concern. The following keywords were applied on the search: ("Peripheral nerve injury") AND (repair OR treatment OR regeneration OR therapy or therapeutics). A total of 2921 publications were downloaded in “plain text” format, with the record content set to “full record and cited references.” After screening, no duplicates were identified. As this study used secondary data, ethical approval was not required.

Bibliometric Analysis

Our bibliometric analysis progressed from a broad overview to detailed insights, encompassing countries/regions, authors/institutions, journals, documents/references, keywords, and research trends, despite the extensive and complex data set. The data were imported into Biblioshiny (R version 4.2.2; Vienna, Austria: Institute for Statistics and Mathematics; www.r-project.org) and VOSviewer version 1.6.18 (Leiden, The Netherlands: Centre for Science and Technology Studies, Leiden University) for further analysis. Biblioshiny was primarily used to visualize and analyze sources, authors, conceptual structure (thematic map), and documents (including author keywords), providing various bibliometric indicators to assess the output of countries, authors, institutions, and journals. Productivity was measured by the number of articles, total citations indicated impact within the academic community, and local citations assessed impact within specific fields. These dimensions are crucial for evaluating research quality. The h-index, which reflects both productivity and impact by counting the number of papers (h) that have been cited at least h times, was also employed.

VOSviewer was utilized to create plots illustrating country and institutional collaboration, as well as mapping co-occurrence, co-citation, and keyword co-occurrence. The following settings were applied: (1) "create a map based on bibliographic data," "read data from bibliographic database files," and "web of science." For the type of analysis and counting method, co-authorship and citation were conducted using the full counting method. The option to "ignore documents with a large number of authors" was selected and capped at a maximum of 25 authors per document. The analysis was performed across the following three units of analysis: authors, organizations, and countries. For author analysis, a minimum of 15 documents was required, resulting in 101 outcomes, with the weight based on the number of documents. For organization analysis, a minimum of 15 documents was needed, yielding 65 outcomes, with the weight based on citations. For country analysis, a minimum of 10 documents was set, producing 33 outcomes, with the weight also based on document citations.

Results

Of the 2929 publications retrieved from the database, seven were retracted publications and one was a publication with an expression of concern, consequently, those eight papers were not included in our analysis. The distribution from 2921 publications was as follows: 84.97% were articles, followed by reviews (14.03%), proceeding papers (2.12%), and other types such as book chapters and early access documents (1.23%). The majority of these publications were written in English (99.17%), with smaller proportions in German (0.34%), Spanish (0.10%), Chinese (0.10%), and other European, American and Asian languages. For our analysis, we included articles published in all languages.

Publication Output and Sources

**Figure 1:**
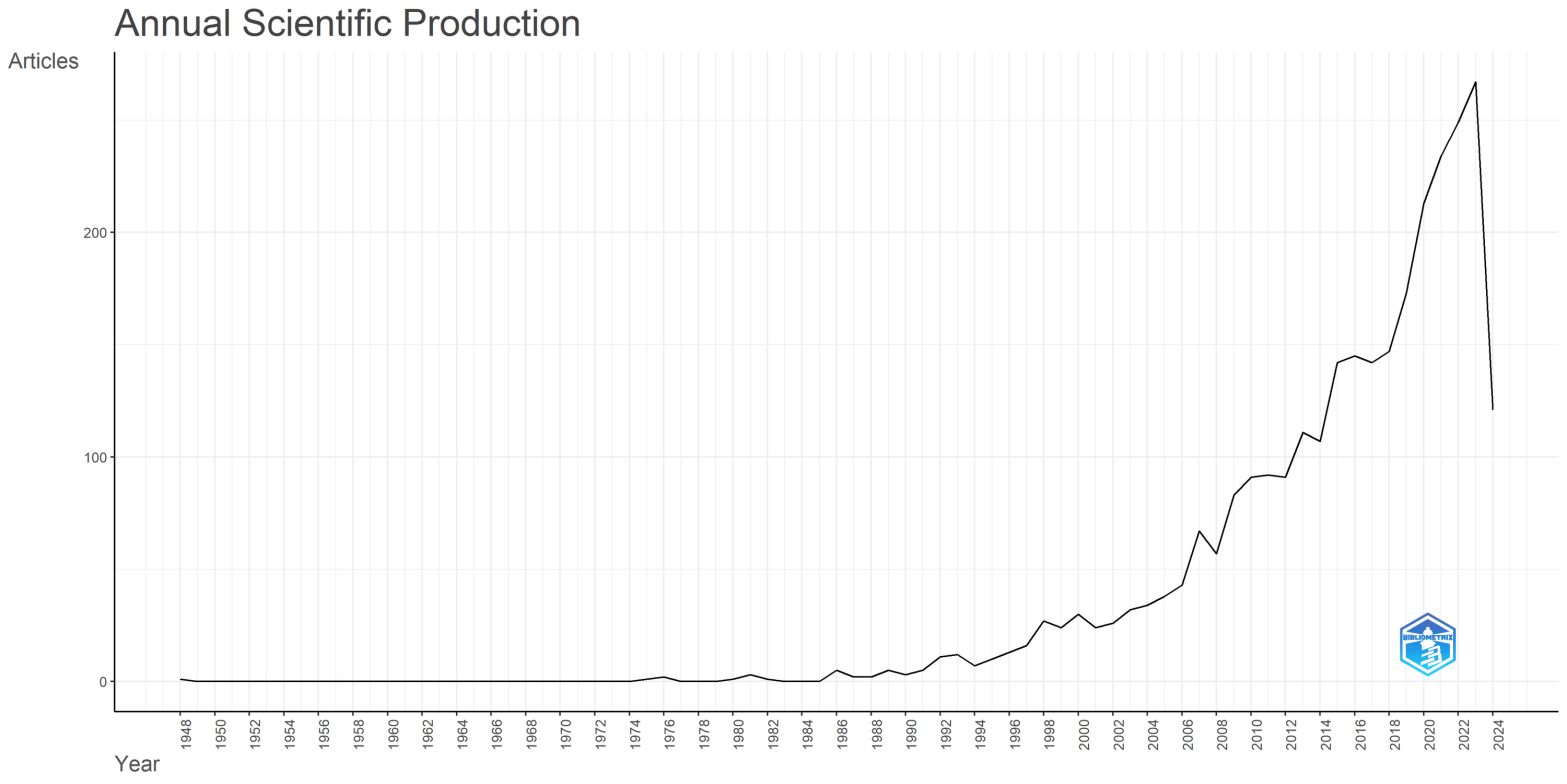
The number of scientific articles published per year from 1984 to 2024. The images are created by the authors of this study using specific software/tools that are indicated in the Materials and Methods section, particularly VOSviewer and Biblioshiny.

**Figure 2:**
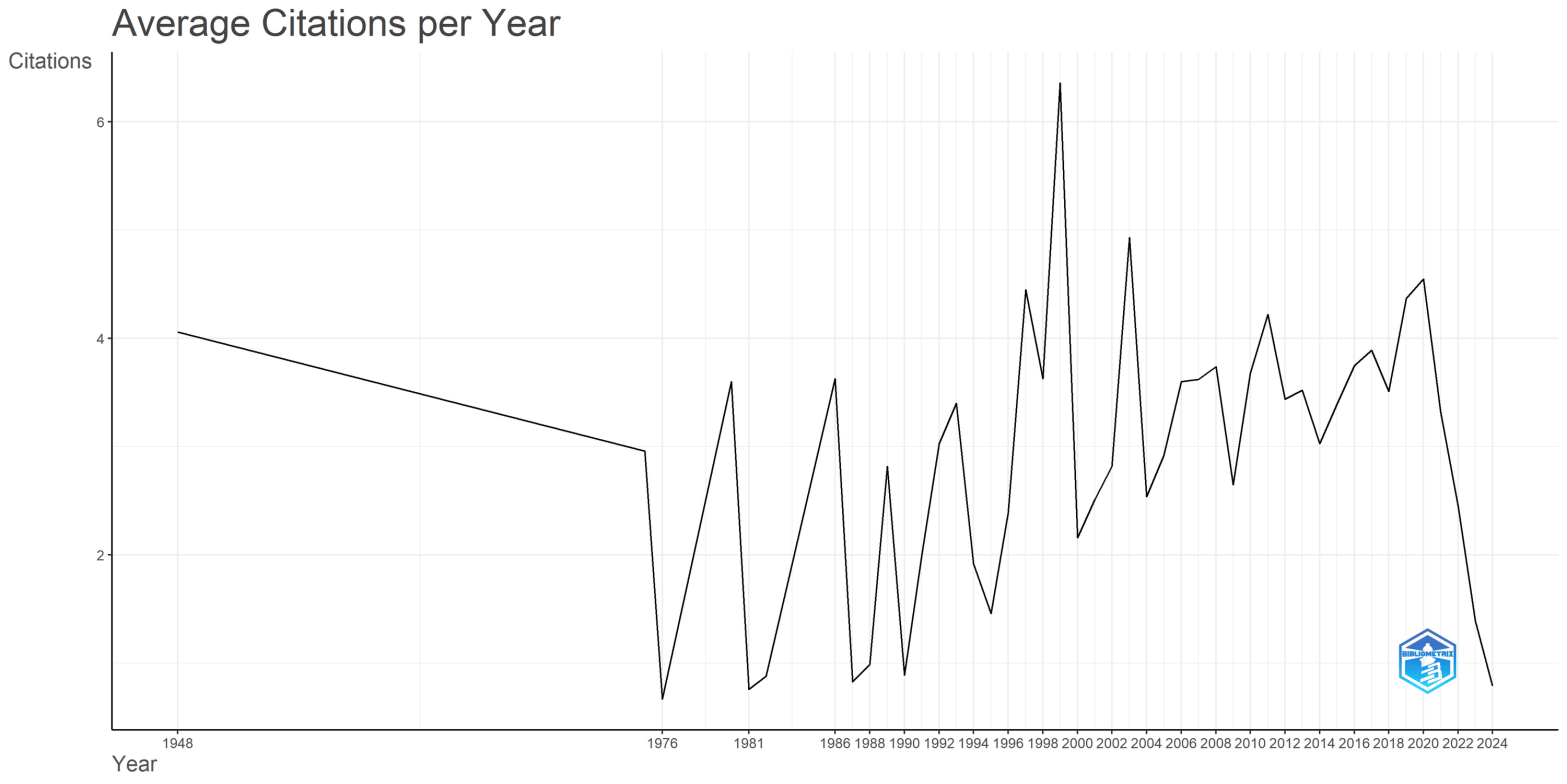
The trend for the average citations per year for published articles on peripheral nerve regeneration research articles from 1948 to 2024. The images are created by the authors of this study using specific software/tools that are indicated in the Materials and Methods section, particularly VOSviewer and Biblioshiny.

Scientific article production remained low until 1991, with minor fluctuations from 1975 to 2006 (Figure 1). Starting around 1992, there was a noticeable increase in output, which became more significant after 2002. The annual number of published articles continued to rise, reaching a peak in 2013, exceeding 100 articles per year. This peak indicates substantial progress and breakthroughs in peripheral nerve regeneration research. After 2016, there was a slight decline in publications, but the trend picked up again from 2018 onward. Figure 2 presents a graph depicting the changes in citation patterns over time, from 1948 to 2024. Initially, between 1948 and the early 1970s, there was a consistent decline in citations, dropping from an average of four citations per year to less than two citations annually. However, starting in 1976, citation rates became more volatile, with noticeable peaks occurring in the late 1980s and early 1990s. Notably, from the early 2000s to 2020, there was an overall increase in average citations, marked by significant peaks around 2003, 2011, and 2020, signifying periods of heightened research impact.

Regarding the most relevant and highly cited journals, "Neural Regeneration Research" had an h-index of 68 with 123 articles, while the "Journal of Neuroscience" stood out with the highest total citation of 6951 (Table 1). Other notable journals included "Experimental Neurology" (h-index 208, 70 articles), "Journal of Neuroscience" (h-index 503, 58 articles), and "Neuroscience" (h-index 246, 46 articles) (Table 2). Analysis of the most highly cited documents in peripheral nerve regeneration research was also performed (Table 3). The top paper, authored by Millan, received 1256 citations, averaging 48.31 citations per year and a normalized citation count of 7.60 [7]. The second most cited paper, by Tsuda et al., had 1212 citations, 55.09 citations per year, and a normalized citation count of 11.18 [8]. Other notable papers are included in Table 3.

**Table 1:** Most cited sources in peripheral nerve regeneration research.

Most cited sources	Total citations
Journal of Neuroscience	6951
Experimental Neurology	4357
Pain	4108
Biomaterials	3045
Brain Research	3032
Proceedings of the National Academy of Sciences of the United States of America	2464
Neuroscience	2320
Neuron	2058
Neuroscience Letters	1957
Nature	1881

**Table 2:** Most relevant sources in peripheral nerve regeneration research.

Sources	H-index	Number of articles
Neural Regeneration Research	68	123
Experimental Neurology	208	70
Journal of Neuroscience	503	58
Neuroscience	246	46
Pain	296	45
Neuroscience Letters	188	37
Journal of Neuroscience Research	177	33
International Journal of Molecular Sciences	269	32
PLoS One	435	32
Brain Research	255	31

**Table 3:** Highly cited papers concerning peripheral nerve regeneration.

Author name and year	Source	Total citations	Total citations per year	Normalized total citations
Millan (1999) [7]	Progress in Neurobiology	1256	48.31	7.60
Tsuda et al. (2003) [8]	Nature	1212	55.09	11.18
Fu and Gordon (1997) [9]	Molecular Neurobiology	942	33.64	7.56
Sindrup and Jensen (1999) [10]	Pain	823	31.65	4.98
Finnerup et al. (2010) [11]	Pain	733	48.87	13.28
Noble et al. (1998) [12]	Journal of Trauma and Acute Care Surgery	687	25.44	7.02
Funakoshi et al. (1993) [13]	Journal of Cell Biology	610	19.06	5.60
Schnell et al. (2007) [14]	Biomaterials	607	33.72	9.31
Gaudet et al. (2011) [15]	Journal of Neuroinflammation	604	43.14	10.21
Malmberg et al. (1997) [16]	Science	543	19.39	4.36

**Figure 3:**
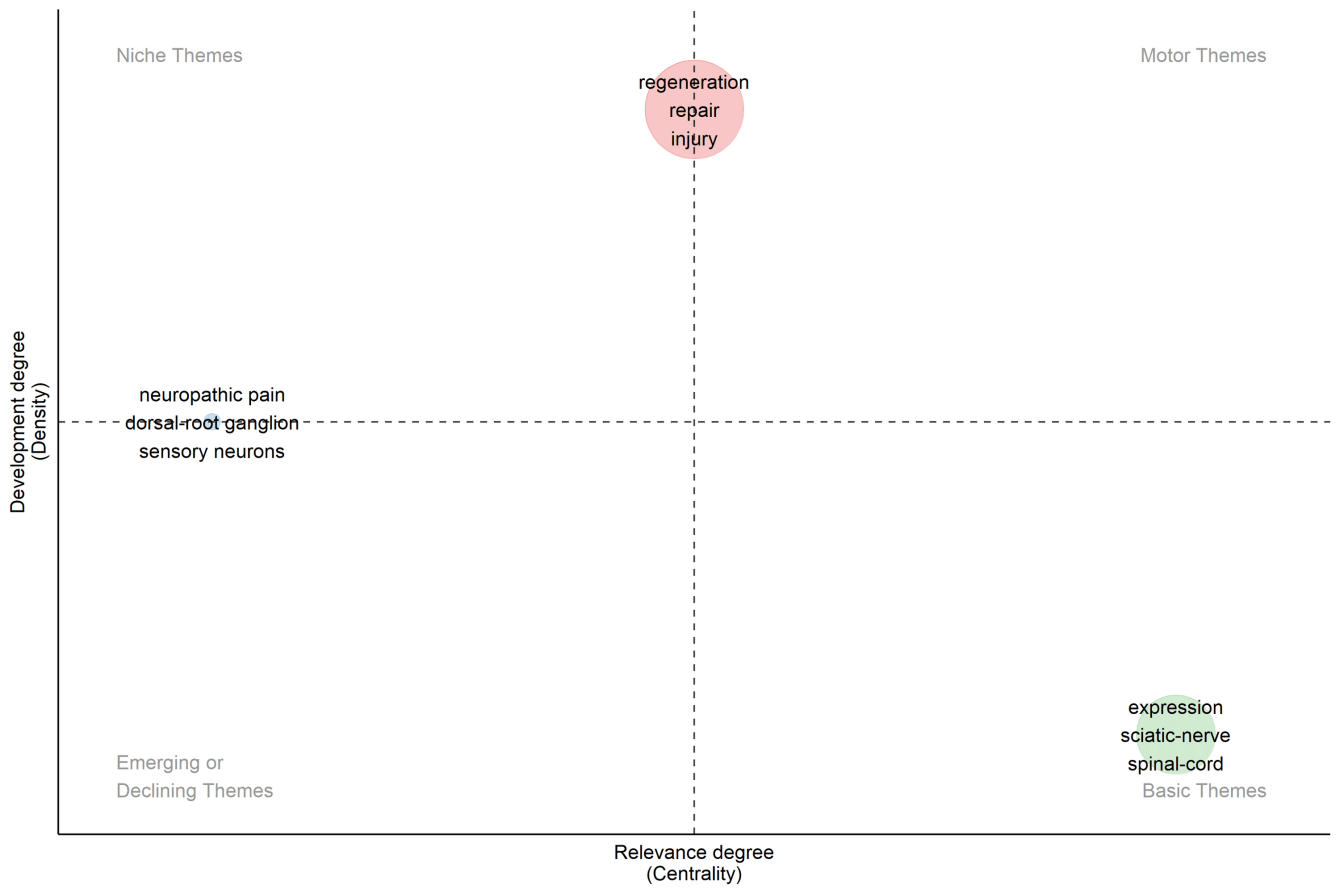
Thematic map illustrating the development and relevance of various research themes in peripheral nerve regeneration, highlighting niche, motor, emerging/declining, and basic themes. The images are created by the authors of this study using specific software/tools that are indicated in the Materials and Methods section, particularly VOSviewer and Biblioshiny.

An analysis of the top 15 keywords revealed that the term "regeneration" appeared the most, with 525 occurrences, followed by "expression" at 402 and "repair" at 357 occurrences. Other notable terms included "injury" (348), "Schwann cells" (347), and "sciatic nerve" (312). Terms related to outcomes and models, such as "functional recovery" (248), "neuropathic pain" (243), and "spinal cord" (215), also featured prominently. Additionally, "axonal regeneration" (207), "peripheral nerve" (192), "growth factor" (185), "rat" (173), "in vitro" (169), and "growth" (160) were significant, indicating a focus on nerve regeneration, injury, and recovery research. The thematic map categorized research themes based on development degree (density) and relevance degree (centrality) (Figure 3). "Regeneration," "repair," and "injury" were identified as central themes with high development and relevance. "Neuropathic pain" was classified as a niche theme with lower centrality but moderate density. "Expression," "sciatic nerve," and "spinal cord" were basic themes, indicating foundational research areas with steady relevance but lower development. “Sensory neuron” was identified as an emerging/declining theme with lower centrality.

Country Scientific Production

**Figure 4:**
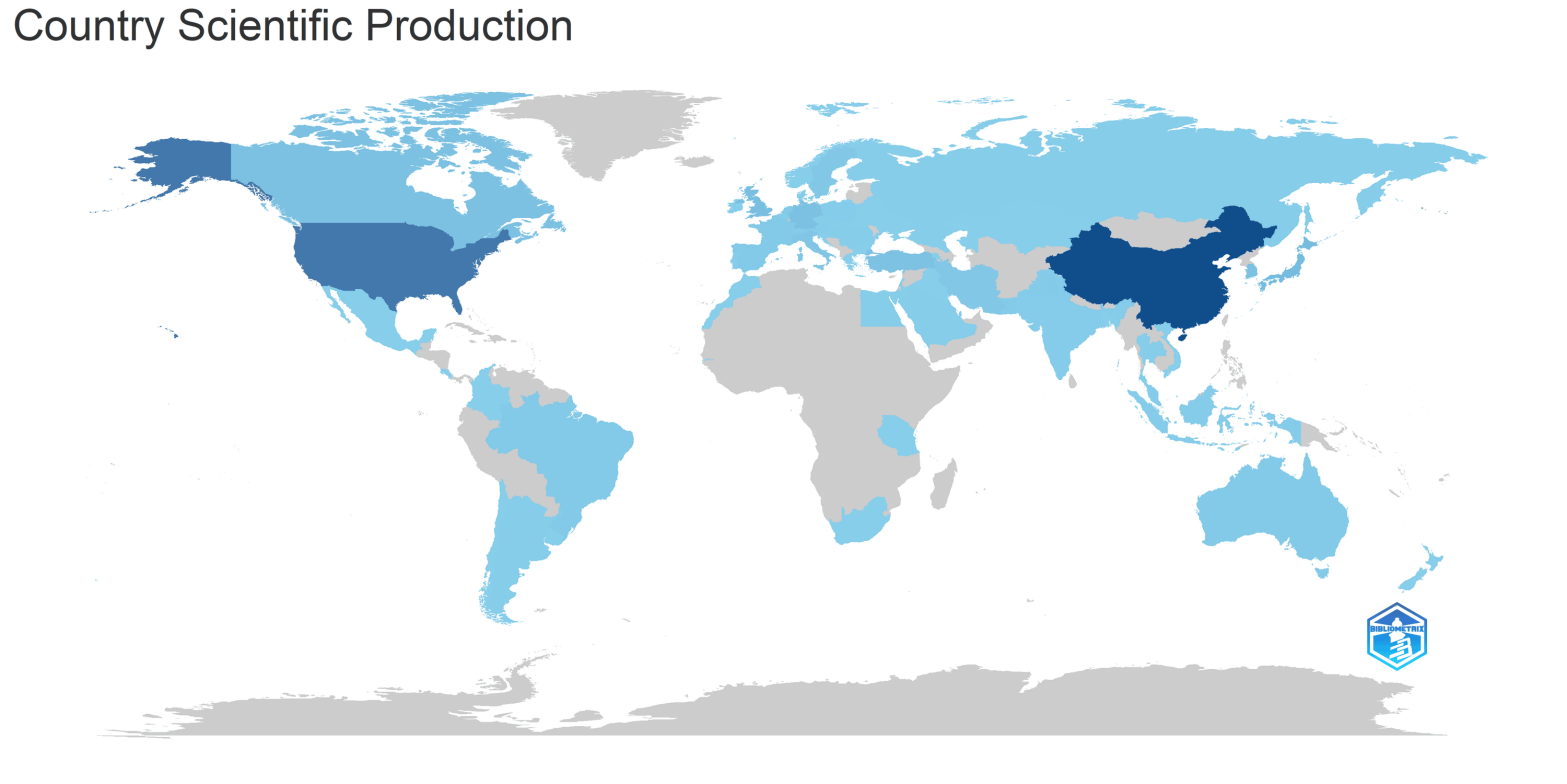
Global distribution of research on peripheral nerve regeneration. Different shades of blue indicate different productivity rates. Dark blue indicates high productivity, light blue indicates low-to-moderate productivity, and gray indicates no articles. The images are created by the authors of this study using specific software/tools that are indicated in the Materials and Methods section, particularly VOSviewer and Biblioshiny.

The analysis of the global distribution of scientific publications revealed that China stood out as the leading producer of scientific output (depicted by the darkest blue in Figure 4). Significant contributions also originated from Canada, Japan, the UK, and the USA (represented in various shades of blue). In contrast, many countries in Africa, parts of Asia, South America, and Eastern Europe (shaded in gray) exhibited lower or negligible scientific output. This map underscored geographical disparities, with the highest research concentrations found in North America, Western Europe, and parts of Asia.

**Figure 5:**
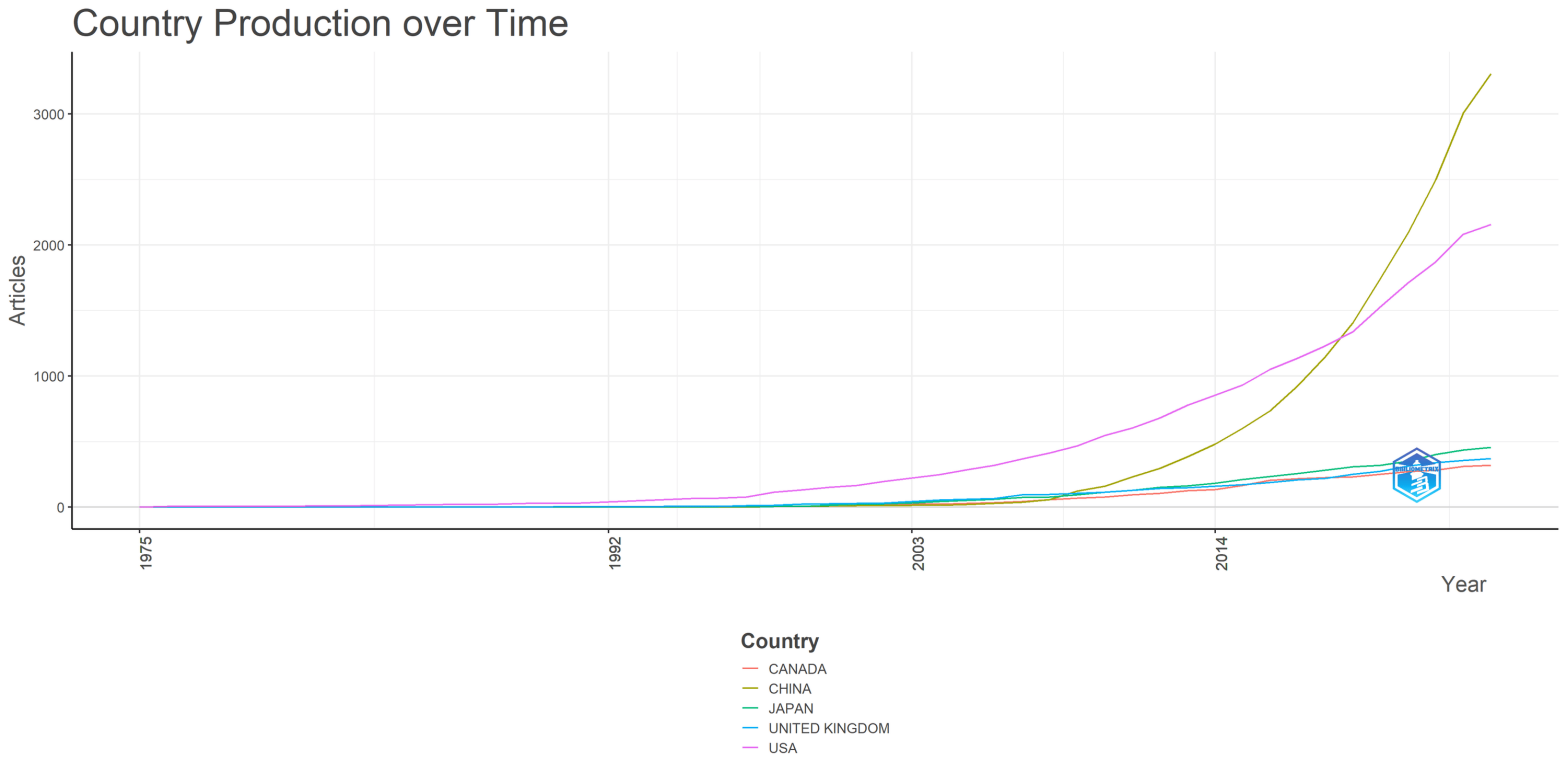
Country production over time of scientific research output related to peripheral nerve regeneration. The images are created by the authors of this study using specific software/tools that are indicated in the Materials and Methods section, particularly VOSviewer and Biblioshiny.

Initially, from 1975 to 2000, all countries had minimal research output (Figure 5). Between 2000 and 2010, there was a gradual increase, with the United States and the United Kingdom slightly leading. However, from 2010 onwards, significant growth was observed, with China showing the most dramatic rise, reaching over 3000 articles by 2023, making it the leading country in this research field. The United States followed, approaching 2000 articles. The United Kingdom showed steady growth, while Japan and Canada also increased their outputs, with Japan slightly ahead. In recent years, the accelerated research output from China and the United States indicates increasing global interest and investment in peripheral nerve regeneration research. Data on the total citations (TC), average article citations, and frequency of articles from different countries showed that the United States received the highest total citations (32550) and frequency (2157) but a moderate average of citations per article (47.60) (Table 4). China had a high frequency of articles (3307) but lower average citations (17.60). Canada, though having fewer articles (320), led to average citations (66.30). France, with 120 articles, had the highest average citations per article (89.90). Other notable countries are shown in Table 3.

**Table 4:** Top 10 countries by citation count and research output in peripheral nerve regeneration.

Country	Total citations	Average article citations	Frequency
USA	32550	47.60	2157
China	17419	17.60	3307
Japan	6448	41.60	458
Canada	6233	66.30	320
United Kingdom	6194	45.20	371
Germany	4265	50.20	314
Sweden	2547	55.40	143
France	2518	89.90	120
Korea	2064	23.20	316
Italy	1923	37.70	192

**Figure 6:**
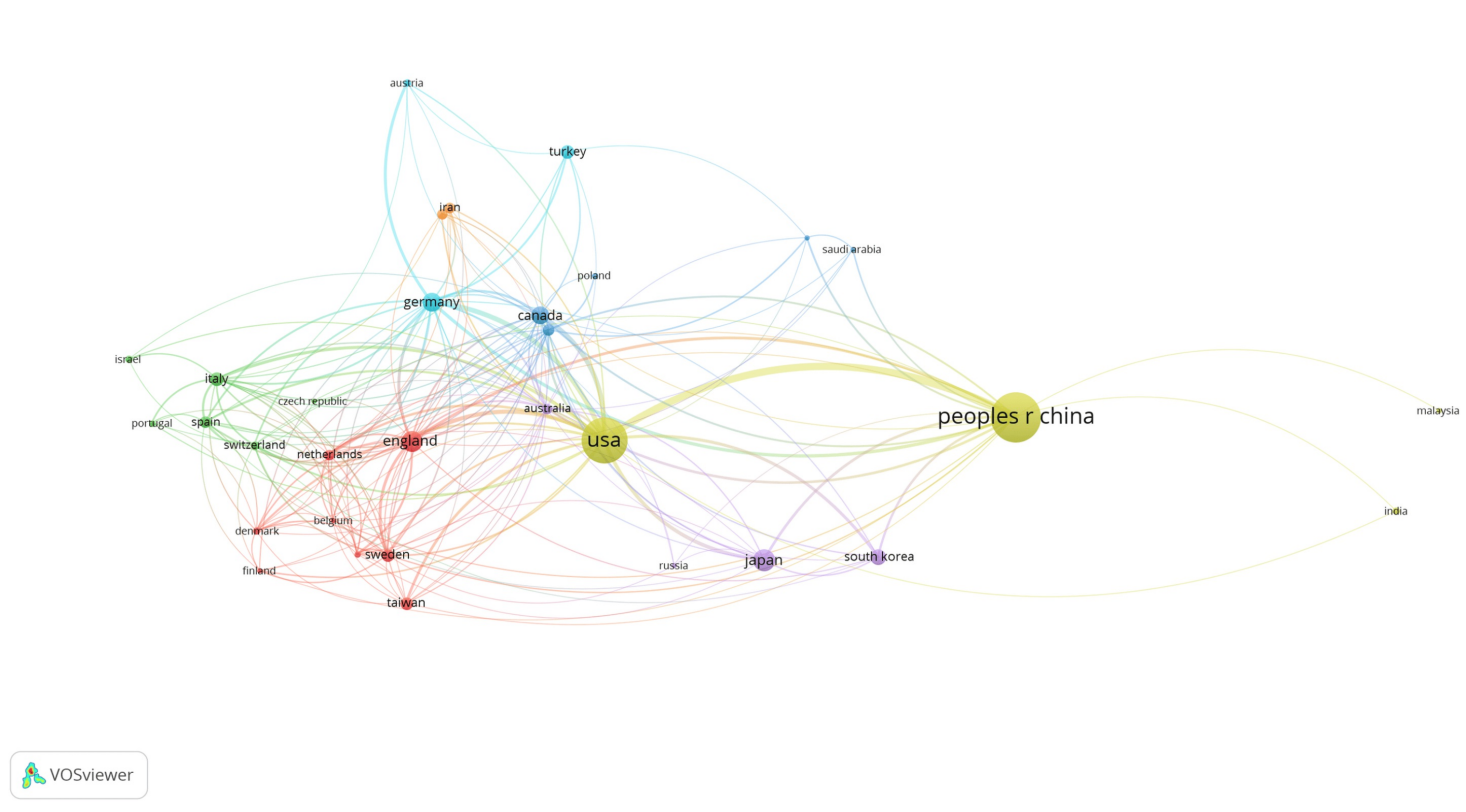
Country collaboration network for peripheral nerve regeneration research. The images are created by the authors of this study using specific software/tools that are indicated in the Materials and Methods section, particularly VOSviewer and Biblioshiny.

**Figure 7:**
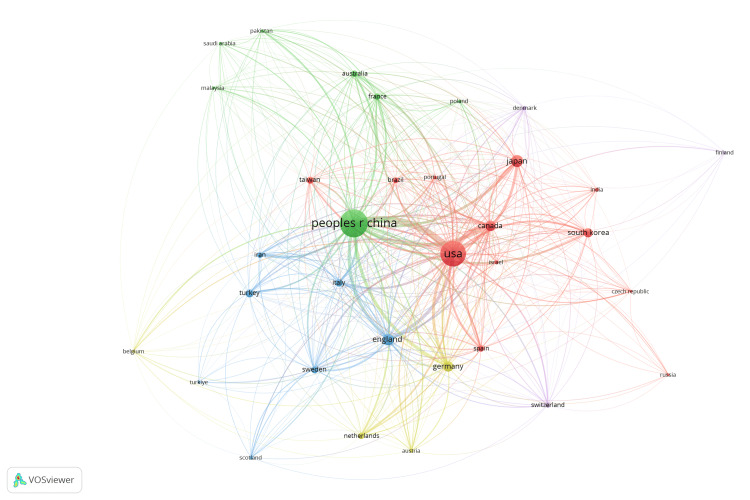
Global citation network in peripheral nerve regeneration research. The images are created by the authors of this study using specific software/tools that are indicated in the Materials and Methods section, particularly VOSviewer and Biblioshiny.

The co-authorship network for peripheral nerve regeneration research, as depicted in Figure 6, highlighted the collaboration patterns among various countries. There were seven clusters, and 194 links from 32 countries, with a total link strength of 817. The United States and China were the most prominent nodes, indicating a significant number of publications and strong collaboration ties. These two countries also served as central hubs connecting with numerous other countries. European nations like Germany, England, and Italy showed strong interconnections, forming a dense cluster, while also maintaining links with the United States and China. Other notable connections included Canada, Japan, and Australia, which played key roles in bridging different clusters. The United States and China emerged as the most significant nodes, indicating a high number of citations and influential research output (Figure 7). There were five clusters, and 410 links from 33 countries, with a total link strength of 11493. These countries were central to the network, with extensive citation links to and from numerous other countries. European countries like Germany, Italy, and England formed substantial clusters, which were interconnected with each other and with the United States and China. Other notable countries in the network included Japan, South Korea, and Canada, which played crucial roles in the global citation network.

Author and Institutional Scientific Production

**Figure 8:**
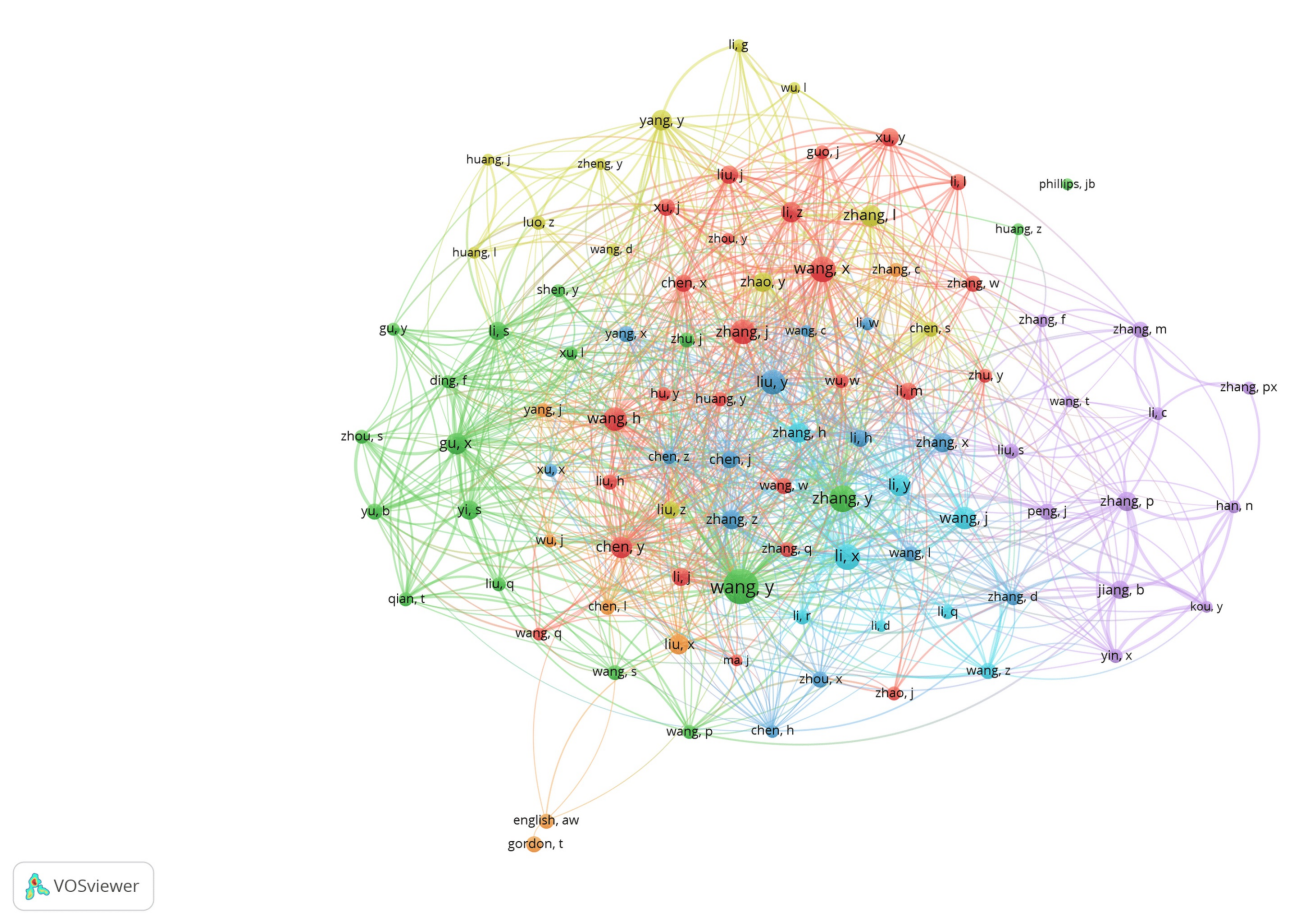
Co-authorship network in peripheral nerve regeneration research. The images are created by the authors of this study using specific software/tools that are indicated in the Materials and Methods section, particularly VOSviewer and Biblioshiny.

**Figure 9:**
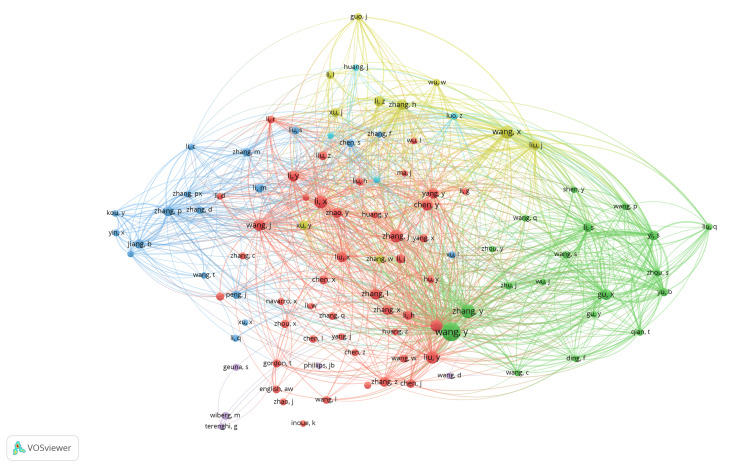
Citation network of authors involved in peripheral nerve regeneration research. The images are created by the authors of this study using specific software/tools that are indicated in the Materials and Methods section, particularly VOSviewer and Biblioshiny.

Top five most relevant authors and affiliations: The most prolific authors included Wang Y from Capital Medical University, who had authored 55 articles, Zhang PX from Peking University with 46 articles, Gu XS from Nantong University with 39 articles, Jiang BG from Peking University People's Hospital with 37 articles, and Yi S from Nantong University with 35 articles. The leading institutions in this research domain were Nantong University, which contributed 356 articles, followed by the University of California System with 153 articles, the University of London with 147 articles, Peking University with 142 articles, and Shanghai Jiao Tong University with 141 articles. Co-authorship collaboration network was densely interconnected, with multiple clusters of researchers frequently collaborating, suggesting a highly collaborative field (Figure 8). There were seven clusters, and 1755 links from 96 countries, with a total link strength of 3619. Prominent nodes, such as those labeled "wang_y," "liu_y," and "zhang_y," indicated key researchers who acted as central hubs within the network. Different colors represented different clusters or groups of researchers who frequently collaborated, highlighting the existence of sub-communities within the larger research network. Citation network analysis identified groups of authors who frequently cited each other (Figure 9). There were six clusters, and 4150 links from 101 countries, with a total link strength of 23408. The thickness of the lines between nodes indicated the frequency of citations between the authors. Prominent clusters were observed, highlighting the interconnectedness and collaborative nature within specific groups. Key authors with significant influence and high citation rates were centrally located with larger nodes, illustrating their pivotal role in the field of peripheral nerve regeneration.

**Figure 10:**
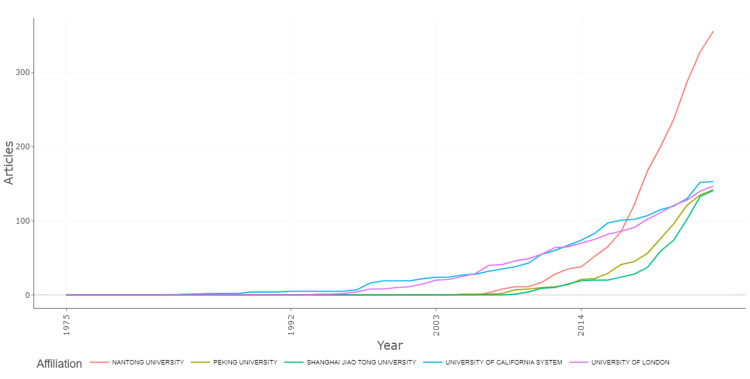
Affiliations' production over time in peripheral nerve regeneration research. The images are created by the authors of this study using specific software/tools that are indicated in the Materials and Methods section, particularly VOSviewer and Biblioshiny.

Regarding the affiliations' production over time for the top five institutions, each university showed a steady increase in research output over time, with notable accelerations starting around the early 2000s (Figure 10). Nantong University exhibited the most growth, particularly after 2014, surpassing the other institutions by a significant margin. The University of California System also showed a consistent upward trend, while Peking University and Shanghai Jiao Tong University followed similar, albeit slightly lower, growth patterns. The University of London showed a steady but comparatively moderate increase in publications. Overall, the graph highlighted a significant rise in research activity across all these institutions. The figure emphasizes the general rise in research activity across these institutions, suggesting that institutions focused on peripheral nerve regeneration have increased their contributions significantly over time, particularly since the early 2000s.

**Figure 11:**
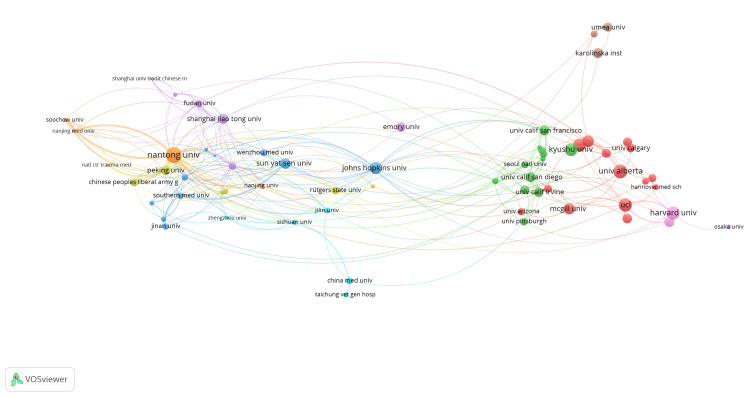
Co-authorship network of institutions in peripheral nerve regeneration research. The images are created by the authors of this study using specific software/tools that are indicated in the Materials and Methods section, particularly VOSviewer and Biblioshiny.

**Figure 12:**
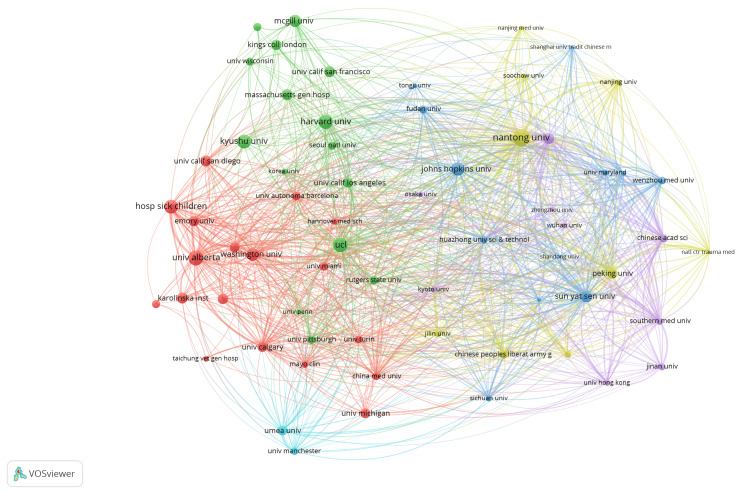
Citation network of institutions involved in peripheral nerve regeneration research. The images are created by the authors of this study using specific software/tools that are indicated in the Materials and Methods section, particularly VOSviewer and Biblioshiny.

A collaboration network analysis of institutions involved in peripheral nerve regeneration research was generated (Figure 11). There were nine clusters, and 246 links from 65 countries, with a total link strength of 469. Major clusters were visible, with prominent institutions such as Johns Hopkins University, Harvard University, and University College London (UCL) acting as central nodes in their respective clusters, indicating high levels of collaboration. The colors of the nodes and edges corresponded to different clusters, highlighting regional and international research partnerships in the field of peripheral nerve regeneration. This interconnected web among institutions from different continents emphasizes the global and collaborative nature of research efforts in advancing the field of peripheral nerve regeneration. The citation network of institutions revealed a densely interconnected landscape with prominent clusters (Figure 12). There were nine clusters, and 246 links from 65 countries, with a total link strength of 469. Key institutions such as Harvard University, Johns Hopkins University, and Nantong University served as central hubs, indicating their significant influence and collaborative relationships within the field. The network showcased a global distribution, with notable contributions from North American, European, and Asian institutions. Strong interconnections between these institutions suggested a robust collaborative environment, facilitating the exchange of knowledge and advancements in peripheral nerve regeneration research.

Discussion

Over the years, extensive research has been conducted to enhance nerve regeneration, exploring various strategies such as surgical interventions, biomaterials, pharmacological agents, and cell-based therapies. Despite the inherent regenerative capacity of the PNS, achieving complete functional recovery remains a challenge. This review and bibliometric analysis aimed to synthesize current advancements in peripheral nerve regeneration and provide insights into research trends and scientific outputs in this field. By evaluating the evolution of research through a bibliometric lens, this study highlighted key developments, influential studies, and emerging areas of interest, offering a comprehensive overview of the progress and ongoing challenges in peripheral nerve regeneration.

Publication Output and Sources

Since 2013, the number of publications in this field has steadily increased, with over 100 articles published annually, indicating a consistent upward trend in research output. Several factors contributed to this growth. Firstly, the quest for effective pharmacotherapy to enhance peripheral nerve healing has gained significant momentum in recent years [6]. Secondly, there is growing recognition of the urgent need for innovative strategies to address nerve injuries, leading researchers to explore the use of artificial nerve guidance conduits and novel biomaterials and agents [5]. In 1999, there was a notable surge in citations within this field. However, subsequent years witnessed a decline in citations, which can be attributed to reduced publication output, resulting in fewer references. Additionally, shifts in research priorities often lead to decreased attention toward older topics as researchers explore new areas. Accessibility also plays a crucial role in mitigating this trend, as articles that are freely accessible or published in open-access journals tend to receive more citations compared to those behind paywalls.

We analyzed the top 10 most globally cited publications. Highly cited studies are generally considered the most important and influential in the field. The most highly cited publication provides a comprehensive overview of the mechanisms involved in the induction of pain. Millan's review highlighted the crucial role of Schwann cells, which support axonal growth by producing neurotrophins like nerve growth factor (NGF), brain-derived neurotrophic factor (BDNF), and glial cell line-derived neurotrophic factor (GDNF), and releasing extracellular matrix molecules [7]. Additionally, Schwann cells release mitogens that promote their own proliferation, creating a conducive environment for nerve regrowth. Tsuda et al. investigated the role of P2X4 receptors (P2X4Rs) in tactile allodynia, a pain hypersensitivity condition often resulting from peripheral nerve injury [8]. Their research identified a significant increase in P2X4R expression in microglia, but not in neurons or astrocytes, following injury to the fifth lumbar (L5) spinal nerve in rats. Fu and Gordon examined how peripheral nerves regenerate after injury, highlighting the crucial roles of neuronal survival, Schwann cell proliferation, and the molecular changes that facilitate axonal growth [9]. Schwann cells create a supportive environment by producing cell adhesion molecules and neurotrophic factors, while macrophages help clear debris through Wallerian degeneration. Despite these processes, complete functional recovery is rare, emphasizing the need for improved strategies to enhance regeneration and repair.

Sindrup and Jensen's study evaluated the efficacy of various pharmacological treatments for neuropathic pain, including conditions like diabetic neuropathy, postherpetic neuralgia, peripheral nerve injury, and central pain [10]. The study concluded that while TCAs remain the most effective treatment for neuropathic pain, other treatments like gabapentin and tramadol may be important due to their better tolerability. Finnerup et al. reviewed the efficacy and safety of various pharmacological treatments for neuropathic pain, including peripheral nerve injury [11]. Lidocaine patches, opioids like tramadol, high-concentration capsaicin patches, and cannabinoids, were found to be effective, albeit at varying levels. Emerging treatments such as botulinum toxin type A showed promise. Despite a substantial increase in published trials, many patients still experience inadequate pain relief, underscoring the need for continued research and improved treatment options. Noble et al. investigated the prevalence, causes, severity, and patterns of associated injuries in patients with limb peripheral nerve injuries at a regional level 1 trauma center [12]. The study analyzed 5777 trauma patients treated at Sunnybrook Health Science Centre from 1986 to 1996, identifying 162 patients (2.8%) with peripheral nerve injuries. The most commonly injured nerves were the radial nerve in the upper extremity and the peroneal nerve in the lower extremity, with motor vehicle crashes being the primary cause.

Funakoshi et al. investigated the role of neurotrophins and their receptors in peripheral nerve regeneration following sciatic nerve injury in adult rats [13]. The focus was on the expression levels of mRNAs for brain-derived neurotrophic factor (BDNF), neurotrophin-3 (NT-3), neurotrophin-4 (NT-4), and their receptors trkA, trkB, and trkC at various time points post-injury. The study concluded that neurotrophins and their receptors exhibit differential regulation following peripheral nerve injury, suggesting a cooperative role in nerve regeneration. Li et al. explored the use of electrospun nanofibers for peripheral nerve regeneration, comparing pure poly-ε-caprolactone (PCL) with a blend of 25% collagen and 75% PCL (C/PCL) [14]. C/PCL fibers significantly improved Schwann cell migration, neurite orientation, and process formation compared to PCL. The study concluded that C/PCL fibers are promising for artificial nerve implants due to their support for cell proliferation and migration.

Gaudet et al. examined inflammatory events after peripheral nerve injury (PNI) and their role in axon regeneration [15]. Unlike the central nervous system (CNS), peripheral nervous system (PNS) axons can regenerate, although this is hindered by nerve gaps and a limited supportive environment. The inflammatory response, driven by Schwann cells and macrophages, clears debris and supports axon growth. In contrast, CNS inflammation often results in scar formation and further damage. Understanding these mechanisms may improve treatments for nerve injuries. Malmberg et al. investigated the role of protein kinase C gamma (PKCγ) in the development of neuropathic pain following peripheral nerve injury [16]. The researchers used PKCγ knockout mice to determine the enzyme's involvement in pain response. They found that these mice showed normal acute pain responses but significantly reduced neuropathic pain behaviors and neurochemical changes in the spinal cord after nerve injury. The study concluded that PKCγ is crucial for the development of neuropathic pain, suggesting it as a potential target for therapeutic intervention in persistent pain conditions.

Researchers strive to have their work reach a wide and engaged audience, often by publishing in journals with a high Journal Impact Factor (JIF) that aligns with their research focus. For peripheral nerve regeneration research, Neural Regeneration Research stands out as the most influential journal, boasting 123 publications. Experimental neurology follows with 70 publications. Interestingly, the most cited documents in this field are spread across various journals, providing researchers with a broad range of reputable publishing options. Additionally, the journal PAIN, established in 1975, has published two of the most cited articles and covers research and reviews in anesthesiology and clinical neurology.

Keywords Analysis

In bibliometric research, publication keywords serve as fundamental elements for representing knowledge concepts and are widely used to uncover the knowledge structure within research domains [17]. Keywords and their co-occurrence analysis are pivotal in bibliometric studies. They offer insights into the main themes and topics of publications, helping researchers grasp the research focus and identify pertinent studies [18,19]. The keyword "regeneration" appeared most frequently, with 525 occurrences, followed by "expression" with 402 occurrences. The significance of Schwann cells in peripheral nerve regeneration placed this keyword in the fifth position. Under pathological conditions, Schwann cells are essential for promoting peripheral nerve regeneration and restoring function [20]. Unsurprisingly, the term "rat" is among the top 15 keywords. Rats have a brachial plexus structure very similar to humans [21], and experimental results using rodent forelimb models are often translated to clinical practice [22]. However, it's important to note that rodents have a faster regeneration capacity compared to humans, which explains why rats are the most commonly used laboratory animals in nerve regeneration studies [23,24]. "Regeneration," "repair," and "injury" were identified as central themes with high development and relevance, highlighting the ongoing efforts toward curing peripheral nerve injuries.

The following 15 keywords can be grouped into categories based on their meaning and interpretation: (1) core concepts of healing and recovery with terms that focus on the biological processes of healing, particularly nerve regeneration and functional recovery - regeneration (525 occurrences), repair (357 occurrences), functional recovery (248 occurrences), axonal regeneration (207 occurrences), growth (160 occurrences); (2) biological processes and molecular studies and these keywords indicate studies focused on molecular mechanisms and gene expression linked to nerve repair and regeneration - expression (402 occurrences), growth factor (185 occurrences); (3) injury and related pathologies and this group highlights the focus on nerve injuries and the associated pathological conditions such as pain - injury (348 occurrences), neuropathic pain (243 occurrences); (4) cellular and tissue models with terms that indicate research centered on specific cell types involved in nerve repair and regeneration - Schwann cells (347 occurrences), sensory neuron (emerging/declining theme); (5) anatomical focus and models with terms that reflect the anatomical areas of focus in nerve injury and regeneration studies - sciatic nerve (312 occurrences), spinal cord (215 occurrences), peripheral nerve (192 occurrences); (6) experimental models with keywords that signify the commonly used experimental models (animal and laboratory studies) in the research - rat (173 occurrences), in vitro (169 occurrences). In summary, the keywords primarily center around nerve regeneration, injury, and recovery, with molecular and cellular mechanisms playing a central role. Additionally, animal models and specific anatomical regions like the sciatic nerve and spinal cord are frequently studied. Thematic mapping further indicates that terms like "regeneration" and "repair" are highly developed and central to this research field, while themes like "neuropathic pain" and "sensory neuron" reflect more niche or emerging areas.

Country and Institutional Scientific Production

Until 2020, the USA led the field of peripheral nerve regeneration with the highest production output. However, by 2023, China experienced a remarkable surge in research activity, becoming the leading country as of July 2024. China also assumed a significant role in international collaborations. The number of citations and average citations per article serve as indicators of an article’s quality, with high-impact and high-quality articles generally receiving more citations. In this regard, the United States ranked first, with a total citation count (TC) of 32550 and an average of 47.60 citations per article. Given China’s high publication volume, four of the top ten institutions in this field were from China, and four were from the United States. Although the UK ranked fourth in terms of publication numbers, it had two institutions in the top 10 list. Despite Japan being third in publication output, it did not have any institutions in the top ten. Nantong University in China emerged as the most active institution in this research domain. China’s increasing focus on peripheral nerve regeneration can be attributed to the high prevalence of peripheral nerve injuries in the country. Approximately 20 million people in China suffer from these injuries, with an additional two million cases annually [25]. These injuries often result in severe disabilities and dysfunctions, hindering individuals’ ability to live or work independently and imposing a significant burden on their families and the national economy [25]. Consequently, addressing peripheral nerve injury (PNI) has become a critical public health concern in China.

China and the United States were the leading countries in peripheral nerve regeneration research, boasting the strongest collaborative networks between their institutions. China also showed significant cooperation with other Asian countries, including Japan, South Korea, India, and Malaysia. The UK actively collaborated with the United States, China, and various European countries such as Sweden, Denmark, Poland, and France. Several research institutions exhibited robust cooperative relationships in this field. For instance, Chinese institutions like Nantong University, Peking University, and Shanghai Jiao Tong University have established strong collaborative ties within the country. These collaborations are often driven by the sharing of resources such as funding, technology, equipment, and facilities, thereby enhancing efficiency and effectiveness. In Japan, Tokyo Medical and Dental University (TMDU) maintains strong partnerships with institutions in the United States, leveraging Japan’s national study abroad programs to support young researchers in gaining experience at US research institutions. However, despite these collaborative efforts, the overall breadth and intensity of cooperation between institutions remain less than ideal. To maintain sustainable international collaboration, it is essential to have a research policy that is both flexible and stable [26].

Author Scientific Production

Among the top 10 authors in the field of peripheral nerve regeneration, Wang Y stood out with 55 published articles, making him the most prolific author. He was followed by Zhang PX with 46 articles and Gu XS with 39 articles. These three authors have made significant contributions to the field. Wang Y is a Neurologist, affiliated with Capital Medical University in China, with an h-index of 84. The h-index is a comprehensive metric used to assess the quantity and impact of a researcher’s academic output [27]. One of Wang Y’s notable studies, titled “Chitosan degradation products facilitate peripheral nerve regeneration by improving macrophage-constructed microenvironments,” explored how chitooligosaccharides (COS), the degradation products of chitosan, promote peripheral nerve regeneration by modulating macrophage behavior and enhancing the microenvironments at injury sites [28].

Gu Xiaosong, Wang Yongjun, and Li Sheng demonstrated the strongest collaboration networks with other prolific authors in the red cluster (Figure 12). Collaboration is vital as it enhances interaction with specialists from various fields, increases the likelihood of funding, and broadens readership [29]. In a 2014 study, Gu Xiaosong, Wang Yongjun, and other authors from China found that Let-7 miRNAs negatively regulate nerve regeneration by targeting NGF, which reduces Schwann cell proliferation and migration. Inhibiting Let-7 miRNAs boosts NGF secretion, enhances axonal outgrowth, and promotes Schwann cell migration in injured nerves. These findings indicate that Let-7 miRNAs could be potential therapeutic targets for improving peripheral nerve regeneration. In the purple cluster (Figure 12), researchers Gordon Tessa from the University of Alberta (Canada) and Susan EM from Washington University School of Medicine (USA), collaborated on a study funded by various U.S. agencies, including the Department of Defense (DOD), National Institutes of Health (NIH), National Science Foundation (NSF), and the New Jersey Commission on Brain Injury Research. Their research explored the potential of biomedical engineering approaches, including synthetic conduits and neurotrophic factors, in improving nerve repair outcomes [30]. The study had 422 citations, indicating its significant impact and recognition in the scientific community. Our findings indicated that a significant number of authors involved in peripheral nerve regeneration research were from China.

Review of the mechanisms of peripheral nerve regeneration

Biological Processes

Cellular and molecular mechanisms underlying nerve regeneration: Peripheral nerves are susceptible to various forms of damage, including crushing or transection, which can lead to nerve degeneration [4]. However, these nerves also possess the capacity for regeneration, although the process is slow [31]. Upon injury, a sequence of cellular and molecular events is triggered, starting with Wallerian degeneration, which occurs in two phases: an early phase (up to five days) and a later phase (days five to 14) [1]. During the early phase, the damaged nerve region and its distal end detach from the corresponding nerve trunk, undergoing significant cellular changes such as cytoskeletal distention, formation of myelin ovoids, and axonal fragmentation. The influx of Ca^2+^ into the distal nerve stump marks the beginning of Wallerian degeneration at the molecular level [1]. Blocking Ca^2+^ channels can delay axonal degeneration for a few days [1]. Following an increase in intracellular Ca^2+^ concentration, calpains are activated, which catalyze the cleavage of cytoskeletal proteins, including neurofilaments. Recent studies have associated Wallerian degeneration with decreasing NAD^+^ levels in the distal axon and increasing NMN levels due to a block in the active transport of NMNAT2 from the cell body to the axoplasm following injury [1].

Role of Schwann Cells in Nerve Repair

As Wallerian degeneration progresses, neighboring Schwann cells discard their myelin, de-differentiate, and proliferate, forming bands of Büngner that bridge the defective peripheral nerve system [32,33]. Schwann cells, part of the neuroglia, normally surround peripheral nerves, forming the myelin sheath that speeds up impulse transmission and protects axons from damage [34]. Upon injury, these cells are activated by injury signals from damaged axons and loss of axonal contact. This activation involves a transcriptional switch that converts Schwann cells into a pro-repair form capable of secreting neurotrophic growth factors to induce axonal regrowth. They also release chemokines and cytokines to recruit macrophages, which clear cellular debris, including axonal fragments and myelin debris, thus preventing inhibition of the regeneration process [1]. 

Axonal Growth and Guidance

Pro-repair Schwann cells form guidance tubes by changing shape to an elongated, tube-like structure, housing regenerating axons within their lumens. These tubes release growth factors concentrated around the regenerating axon, guiding and coordinating its regeneration while preventing fibroblast permeation and scar formation. These structures persist until axonal regeneration is complete [6,35,36].

Neurotrophic Factors and Their Signaling Pathways

Neurotrophic growth factors, including brain-derived neurotrophic factor (BDNF), play crucial roles in peripheral nerve regeneration by supporting cell survival, proliferation, differentiation, and morphogenesis. These factors maintain a regenerating microenvironment necessary for axonal elongation and sprouting. BDNF, for example, contributes to neurite formation, axonal outgrowth, apoptosis prevention, and formation of chemotactic factors. Transforming growth factor-beta (TGF-β) is involved in phenotypic changes in Schwann cells, immune modulation, intrinsic growth activation, and blood-brain barrier regulation at the injury site [37-39].

Intrinsic and Extrinsic Factors

Genetic factors influencing nerve regeneration: Certain genetic conditions affect nerve regeneration, such as Charcot-Marie-Tooth (CMT) disease, a hereditary peripheral neuropathy. CMT has several forms, including CMT1 (myelin damage) and CMT2 (axon damage). Mutations in genes such as PMP22, MPZ, GJB1, and MFN2 underlie these subtypes, causing structural instability of axons and demyelination, inhibiting axonal regrowth [40].

Influence of the Extracellular Matrix and the Microenvironment

The extracellular matrix (ECM) and microenvironment significantly influence nerve regeneration. Genes coding for ECM molecules, including intercellular adhesion molecules (ICAMs) and galectins, are upregulated during regeneration. The fibrin matrix, when combined with neurotrophic growth factors like BDNF, aids axonal regeneration. Matrix metalloproteinases, such as MMP-7, facilitate Schwann cell migration to the injury site by breaking down the ECM [39,41,42].

Role of Inflammation and Immune Response

Inflammation and immune responses play crucial roles in nerve regeneration. Chemokines and cytokines produced after axonal injury help create a favorable microenvironment for regeneration. Upregulation of various cytokines and chemokines, such as TNF-α, IL-1, IL-6, and IL-10, occurs at different stages post-injury, contributing to macrophage recruitment and debris clearance. IL-10, an anti-inflammatory cytokine, limits pro-inflammatory cytokine secretion and promotes axon regeneration and myelination [39,43].

Current Therapeutic Approaches

Surgical techniques: Autografts are considered the gold standard for nerve reconstruction, particularly in cases where tension-free neurorrhaphy is difficult. According to Lam et al., autografts are favored for their minimal immunological reactions and optimal regenerative microenvironment, especially for long nerve deficits (>3 cm), proximal injuries, and critical nerve injuries [5]. The sural nerve is commonly used as a donor due to its accessibility and compatibility, promoting successful nerve regeneration. However, autografts have drawbacks, including potential donor site morbidity and limited availability, complicating multiple or extensive nerve repairs [5]. Nerve allografts are a favorable alternative due to their availability and supportive properties in nerve regeneration. According to Hussain et al., allografts, sourced from cadavers or donors, retain the endoneurial microstructure and Schwann cells (SCs) from the donor, aiding the regeneration process [44]. Systemic immunosuppression is crucial to prevent graft rejection [44]. Nerve guidance conduits offer an innovative solution for nerve repair, addressing the limitations of autologous grafting and neurorrhaphy. According to Lam et al., these conduits facilitate Schwann cell proliferation and promote organized axon outgrowth while preventing fibrous tissue infiltration [5]. Ideal conduits should be biodegradable, flexible, permeable to trophic factors and metabolic waste, and provide mechanical support with biomimetic properties. Recent advancements in bioengineering have led to improved materials and designs, with natural polymers like collagen and chitosan being popular for their regenerative environment [5]. Direct nerve repair using microsurgical techniques, as described by Hussain et al., is considered the gold standard for treating axonotmesis and neurotmesis, ensuring continuity and endurance between nerve segments [44]. The three main categories of nerve repair techniques include epineurial repair, perineurial repair, and group fascicular repair. Each technique serves a crucial role in optimizing nerve recovery based on the specific nature of the nerve injury and desired functional outcomes [44].

Pharmacological Interventions

The 4-aminopyridine (4-AP) is a voltage-gated potassium channel blocker with reported beneficial effects against various nervous system diseases. According to Hussain et al., 4-AP promotes durable recovery and remyelination following acute traumatic nerve injury, enhancing nerve repair [44]. Quercetin, a flavonoid known for its anti-inflammatory and antioxidant activities, has demonstrated positive effects on nerve regeneration, accelerating the process and shortening recovery periods in mild to moderate nerve injuries [45-47]. Ursolic acid (UA), a pentacyclic triterpenoid with a wide range of beneficial properties, promotes the regeneration of injured sciatic nerves in mouse models [48,49]. Tacrolimus, a macrolide immunosuppressant, significantly enhances peripheral nerve regeneration and accelerates recovery of neurological functions by reducing scar formation. Valproic acid, an anti-epileptic and mood-stabilizing drug, promotes neurite outgrowth by activating the ERK pathway and increasing the levels of bcl-2 and growth cone-associated protein 43 in the spinal cord [44]. Herbal medicines such as Ginkgo biloba and curcumin have shown potential benefits in promoting neuroregeneration due to their antioxidant and anti-inflammatory properties [5,50].

Biological Therapies

Nerve growth factor (NGF) plays a multifaceted role in promoting peripheral nerve outgrowth and differentiation. According to Carvalho et al., NGF's effects vary, promoting both pro-inflammatory and anti-inflammatory responses, essential for tissue repair while reducing excessive inflammation [6]. Brain-Derived Neurotrophic Factor (BDNF), synthesized by Schwann cells, motor neurons, and dorsal root ganglion neurons, promotes nerve regeneration through the activation of the JAK/STAT pathway in Schwann cells [6]. Bone marrow stromal stem cells (BMSCs) produce and secrete various neurotrophins, enhancing peripheral nerve repair. According to Kubiak et al., BMSCs have been successfully tested as supplements to nerve scaffolds [51]. Adipose-derived stem cells (ADSCs) show significant promise in nerve repair by producing several growth factors and supporting neurite response and myelination of DRG neurites in vitro [51]. Neural crest stem cells (NCCs), originating during embryological development, present strategic potential for nerve repair due to their multipotency [51]. Amniotic mesenchymal stromal cells (AMSCs) outperform other stem cells in nerve repair, significantly enhancing electrophysiologic and functional recovery [51]. Umbilical cord-derived mesenchymal stem cells, particularly those from Wharton's jelly and umbilical cord blood, have been studied for their therapeutic potential in various nerve injuries [51]. Gene therapy involves introducing a therapeutic gene into cells to treat disease, with viral vectors being the most efficient approach. According to de Winter et al., these vectors have shown promise in expressing transgenes in primary sensory or spinal motor neurons for nerve regeneration [52]. Initial studies on rats have also shown that the administration of melatonin after peripheral nerve surgery was effective in reducing the formation of neuromas and the elevation of collagen content on the junction site, in addition to reducing the immunoreactivity of the repair region [53,54]. Thus, new papers have also mentioned the possible role of melatonin in peripheral nerve degeneration diseases [54].

Physical and Rehabilitation Therapy

Electrical stimulation (ES) has shown significant benefits in tissue regeneration, including peripheral nerves. According to Maeng et al., SP-ES (single-prolonged electrical stimulation) has been validated in animal studies for promoting axon regeneration after nerve injury [55]. Exercise promotes axonal growth and phenotypic changes in peripheral nerve architecture. According to Maugeri et al., aerobic exercises like swimming and walking are particularly effective in treating peripheral nerve injuries [56].

Emerging and Experimental Approaches

Nanotechnology and biomaterials: Nanotechnology and nano-based materials have garnered significant attention among researchers over the past three decades. Nanoparticles, due to their advantageous size, serve as links between the molecules of various composite polymers, directly influencing the nanocomposite's thermal, mechanical, electrical, catalytic, optical, and chemical properties [57]. There is great potential for bio-nanocomposite materials to mimic the characteristics of native extracellular matrix (ECM). Natural ECM proteins, such as collagen and laminin, exhibit specific nano-structural features. One of the most promising nanomaterials for medical applications is polyhedral oligomeric silsesquioxane (POSS) nanoparticles. Their unique chemical composition allows them to potentially improve the physiochemical properties of copolymers [57]. Incorporating POSS nanoparticles into polycaprolactone (PCL) has resulted in the synthesis of POSS-incorporated poly(caprolactone) urea/urethane (POSS-PCL) nanocomposite polymer, which demonstrates significantly enhanced physiochemical properties, including increased tensile strength and surface roughness, compared to conventional PCL [57].

Nanofibrous scaffolds hold significant potential in neural tissue engineering due to their ability to mimic native tissue tubular structures, including axons, microtubules, and ion channels [57]. These scaffolds can be fabricated using various techniques such as electrospinning and self-assembly with materials including synthetic polymers, proteins, lipids, DNA, and glass. Several processing parameters such as solution flow rate, applied voltage, polymer concentration and molecular weight, and the distance between the needle tip and the ground collection plate can directly or indirectly affect the properties of nano-fibrous scaffolds. This adaptability enhances tissue regeneration by enabling the coating of the nano-fiber surface with various biochemical substances essential for cell survival, growth, and differentiation [57].

Tissue Engineering

Three-dimensional printing and bioprinting are computer-driven rapid prototyping techniques that have proven to be valuable tools in developing polymer-based scaffolds for neural regeneration. These technologies provide high precision and control in replicating structures that match the architecture of native nerve tissue [58]. Using computer-aided design (CAD) software, these methods generate blueprints to guide conduit assembly with the desired spatial arrangement of printing materials. Stereolithography (SL) is the most widely used rapid prototyping technique for developing nerve guidance conduits (NGCs), followed by extrusion and inkjet printing. All these methods build 3D objects layer-by-layer, but they differ in how the material is processed during printing [58]. The standard SL microfabrication system can be modified to print an entire 3D object in a single step by photopolymerizing a 2D cross-sectional projection of the 3D structure onto a single resin layer with the desired object thickness. This process is known as directed mirror device (DMD) SL printing [58].

Advanced Imaging and Diagnostic Techniques

Current state-of-the-art techniques for monitoring nerve regeneration in humans, such as electromyography and nerve conduction studies, have limitations including poor localization, operator-dependent variations, ambient temperature effects, and patient age variability. Advanced MR imaging techniques like diffusion tensor imaging (DTI) and diffusion-weighted imaging (DWI) are based on the movements of water molecules within biological tissues along the field gradient [59]. Measuring diffusion gradients along six noncollinear directions allows for the delineation of the fractional anisotropy (FA) of nerve tissues. FA values and changes in diffusivity, measured as the diffusion coefficient (DC), can serve as sensitive indicators of nerve fiber integrity or damage after peripheral nerve injury (PNI). Reliable, safe, noninvasive, repeatable, and reproducible submicron noninvasive technologies can help objectively determine the nature or degree of PNI, identifying and localizing changes in nerve anatomy such as edema, myelin debris, or fascicular/axonal alterations [59].

The development and validation of advanced noninvasive imaging modalities and multipronged strategies, such as molecular or cellular nanoimaging with near-infrared (NIR) fluorophores or multimodality imaging with positron emission tomography (PET)/MRI, hold tremendous promise. These approaches can objectively diagnose nerve injury, monitor nerve regeneration longitudinally, assess treatment responses to neurotherapeutics, and inform timely and accurate treatment strategies [59]. Advanced imaging techniques like MRI, CT, and ultrasound are excellent for visualizing anatomical features. MRI and ultrasound are particularly useful in neurological research because they provide morphological information on peripheral nerve size and fascicular organization [60]. CT, however, has no significant role in visualizing the structure or anatomy of the nervous system due to inadequate sensitivity in soft tissue contrast and concerns about radiation exposure. In clinical applications, ultrasound is affordable, safe, and non-invasive, and patients prefer it to MRI [60].

MRI analysis is consistent with current findings. For example, Liao et al. evaluated longitudinal changes in nerve healing in rats following tissue-engineered construct implantation using MRI [61]. They discovered that nerves implanted with mesenchymal stem cell (MSC)-seeded tubes had better functional recovery and nerve regeneration, but had a slower return to baseline T2 relaxation time and a faster decline in gadofluorine M enhancement than nerves implanted with chitosan tubes alone. Nonetheless, there is a lack of data on the efficacy of transplanted stem cells in treating acute peripheral nerve damage [61].

This study has some limitations. It relies solely on the Web of Science Core Collection, which may omit relevant research from other databases, leading to selection bias. The analysis is predominantly based on English-language publications, potentially excluding significant non-English studies. While the analysis aimed to be comprehensive by including all languages, the relative scarcity of non-English studies in the dataset (as reflected in the analysis) could still lead to underrepresentation of non-English research. To fully address this, it would be beneficial to explore non-English databases more extensively or include more balanced multilingual contributions. Citation and publication trends may be influenced by changing research priorities and accessibility issues, impacting the long-term visibility of earlier work. Additionally, the inherent focus on quantitative metrics in bibliometric analysis might overlook qualitative research impact. Geographical disparities in research output and less-than-ideal collaboration networks could limit the global applicability and knowledge exchange in this field. These factors should be considered when interpreting the study's findings and conclusions.

## Conclusions

This comprehensive review and bibliometric analysis revealed the significant advancements made in the field of peripheral nerve regeneration over recent decades. Despite the inherent regenerative capabilities of the peripheral nervous system, achieving complete functional recovery following nerve injuries remains an ongoing challenge. The analysis revealed that research output in this domain has been steadily increasing, particularly from leading countries such as China and the United States, which have demonstrated a strong commitment to advancing knowledge and therapeutic strategies in this field. Key themes identified included the crucial role of Schwann cells in facilitating axonal regrowth, the exploration of various surgical techniques and biomaterials, and the promising potential of emerging therapies such as gene therapy, stem cell applications, and nanotechnology. The collaborative networks and influential studies highlighted in this analysis provide a solid foundation for future research, which must continue to innovate and refine approaches to enhance nerve repair and regeneration. Ultimately, while significant progress has been made, the journey toward achieving complete functional recovery for patients with peripheral nerve injuries demands sustained research efforts, interdisciplinary collaboration, and the translation of laboratory findings into effective clinical applications.
